# Antifungal activity of amphotericin B and voriconazole against the biofilms and biofilm-dispersed cells of *Candida albicans* employing a newly developed *in vitro* pharmacokinetic model

**DOI:** 10.1186/s12941-015-0083-3

**Published:** 2015-04-03

**Authors:** Mohamed El-Azizi, Noha Farag, Nancy Khardori

**Affiliations:** German University in Cairo, GUC, Faculty of Pharmacy and Biotechnology, Department of Microbiology, Immunology and Biotechnology, Al-Tagmoa Al-Khamis, New Cairo City, Egypt; East Virginia Medical School, Virginia, USA

**Keywords:** *Candida albicans*, Biofilm, Amphotericin B, Voriconazole, Pharmacokinetic biofilm model

## Abstract

**Background:**

*Candida albicans* is a common cause of a variety of superficial and invasive disseminated infections the majority of which are associated with biofilm growth on implanted devices. The aim of the study is to evaluate the activity of amphotericin B and voriconazole against the biofilm and the biofilm-dispersed cells of *Candida albicans* using a newly developed *in vitro* pharmacokinetic model which simulates the clinical situation when the antifungal agents are administered intermittently.

**Methods:**

RPMI medium containing 1–5 X 10^6^ CFU/ml of *C. albicans* was continuously delivered to the device at 30 ml/h for 2 hours. The planktonic cells were removed and biofilms on the catheter were kept under continuous flow of RPMI medium at 10 ml/h. Five doses of amphotericin B or voriconazole were delivered to 2, 5 and 10 day-old biofilms at initial concentrations (2 and 3 μg/ml respectively) that were exponentially diluted. Dispersed cells in effluents from the device were counted and the adherent cells on the catheter were evaluated after 48 h of the last dose.

**Results:**

The minimum inhibitory concentration of voriconazole and amphotericin B against the tested isolate was 0.0325 and 0.25 μg/ml respectively. Amphotericin B significantly reduced the dispersion of *C. albicans* cells from the biofilm. The log_10_ reduction in the dispersed cells was 2.54-3.54, 2.30-3.55, and 1.94-2.50 following addition of 5 doses of amphotericin B to 2-, 5- and 10-day old biofilms respectively. The number of the viable cells within the biofilm was reduced by 18 (±7.63), 5 and 4% following addition of the 5 doses of amphotericin B to the biofilms respectively. Voriconazole showed no significant effect on the viability of *C. albicans* within the biofilm.

**Conclusion:**

Both antifungal agents failed to eradicate *C. albicans* biofilm or stop cell dispersion from them and the resistance progressed with maturation of the biofilm. These findings go along with the need for removal of devices in spite of antifungal therapy in patients with device-related infection. This is the first study which investigates the effects of antifungal agents on the biofilm and biofilm-dispersion of *C. albicans* in an *in vitro* pharmacokinetic biofilm model.

## Background

Fungal infections are most commonly caused by Candida spp., particularly *C. albicans*, *Candida tropicalis* and *Candida parapsilosis* [1,2]. These yeasts are opportunistic pathogens that are capable of causing a variety of superficial and invasive disseminated infections [3]. Their emergence as important nosocomial pathogens is associated with modern medical procedures, such as the use of immunosuppressive and cytotoxic drugs, broad spectrum antibiotics, and the use of central venous catheters as well as implanted devices of various kinds [4,5]. Data from the US National Health Care Safety Network (NHSN) rank these Candida species as the fourth most common cause of bloodstream infection, behind coagulase-negative staphylococci, *Staphylococcus aureus* and enterococci [6] with mortality rate approaching 40% [7].

Among *Candida* species, *C. albicans* is the most common cause of invasive infections. It is the leading cause of disseminated fungal infection in neonates, immunocompromised hosts, diabetics, and postoperative patients [8].

Candida species resistant to antifungal agents have been reported worldwide [9]. Biofilm formation by Candida species and resistance to antifungal agents are important factor in their contribution to human disease.

Recent evidence suggests that the majority of disease produced by *C. albicans* is associated with biofilm growth [10]. *C. albicans* is the fourth and third leading cause of hospital-acquired bloodstream and urinary tract infections, respectively [11]. Up to 70–80% of Candida bloodstream infections are associated with central venous catheters, and the majority of Candida urinary tract infections are associated with indwelling urinary catheters [12]. Candida biofilms were found to be resistant to clinically important antifungal agents including amphotericin B and azoles [13,14].

*In vivo* intermittent administration of antibiotics usually results in incremental decline in the antibiotic levels in serum and tissues with the microorganisms being exposed to both supra- and sub- minimum inhibitory concentrations (MICs) during the dosing interval. Therefore, *in vitro* experiments that subject microorganisms to constant levels of antibiotics do not reflect the true *in vivo* interaction [15]. Several *in vitro* kinetic models have been constructed to simulate the serum antibiotic concentration-time curve obtained in humans [15]. These models are mainly used to study the microorganisms in the planktonic phase while few of them were developed to study bacterial biofilms [16,17].

The aim of the present study is to investigate the antifungal activity of amphotericin B and voriconazole against the biofilm and the biofilm-dispersed cells of *C. albicans* in an *in vitro* model that mimics the *in vivo* interactions when the drugs are administered intermittently. For this purpose, a new pharmacokinetic biofilm model, which uses a novel biofilm device, was developed. This is the first study which investigates the effects of antifungal agents on biofilm and the dispersed biofilm cells of *C. albicans* in a pharmacokinetic model.

## Materials and methods

Unless otherwise indicated, all chemicals (analytical grade) were purchased from Sigma-Aldrich, Saint Louis, Missouri, USA.

### Antifungal

Amphotericin B (Amp-B) was purchase from Sigma-Aldrich, Saint Louis, Missouri, USA. Voriconazole (VCZ) was kindly provided by Pfizer, United Kingdom.

### Microorganism

A clinical isolate of *C. albicans* (CA04)*,* isolated from blood of a patient with central venous catheter, was used in this study. The isolate was identified to species level by using API 20 C AUX for yeast, Bio Merieux, Vitek Inc., Hazelwood, Missouri, USA.

### *In vitro* susceptibility of *C. albicans* to Amp-B and VCZ

The susceptibility of the isolate to Amp-B and VCZ was determined by broth microdilution method described by the Clinical and Laboratory Standards Institute (CLSI) [18]. After determining the MIC, the minimum fungicidal concentration (MFC) of each drug required to kill 99% of the yeast cells was determined by spreading of 10 μl portions from each well with no growth on Sabouraud Dextrose Agar plates. Following incubation for 24 hours, the plates were observed for growth and the MFCs were calculated.

### *In vitro* biofilm device

**Figure 1:**
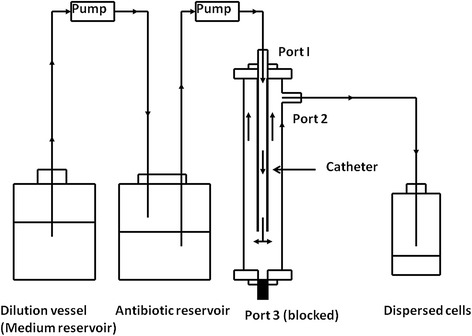
**A novel** ***in vitro*** **biofilm device integrated in a pharmacokinetic biofilm model system.** The device was configured to simulate the *in vivo* condition in which the biofilms of C*. albican* on IV vascular catheter are under continuously perfusion with fresh medium and exposed to exponentially decreasing concentrations of antifungal agents.

A novel *in vitro* biofilm device was developed (Figure 1). The device comprises a tubular body defining a test chamber. The size of the test chamber is of 10 cm height and 0.80 cm diameter with a volume of 5 ml. The body has upper and lower ends provided with closures. Each of the ends has one port which can be used as an outlet or inlet based on the study conditions. The body has a built in side port and all the three ports can be connected to a tubing system or blocked by the removable closures. The ports in the upper and lower ends of the device are designed to mount the tested materials (catheters or tubes). The design allows the fluid to be pumped through the inner lumen of the implant tube before filling the inner chamber to allow biofilm formation on the inner and outer surfaces of the catheters.

The various parts of the device may be fabricated from stainless steel, Pyrex glass or other materials which do not affect the formation of the biofilm.

The design of the device used in this study permits low laminar flow system with very low shear stress on the inner and outer catheter surfaces.

### Pharmacokinetic biofilm model in a dynamic cell flow system

The device was configured to simulate the *in vivo* condition in which microorganisms are exposed to supra and sub MIC of the antifungal agents following intermittent administration of the drugs as in clinical practice (Figure 1). The system is a typical dynamic cell flow system in which antifungal agent can be delivered to the biofilm in sequence by two intra venous (IV) infusion pumps. The first pump delivers fresh medium at a specific flow rate to a second container which contains the antimicrobial agent at a concentration equivalent to its C_max_ (The peak concentration of a drug observed after its administration in the human body). A second pump is used to deliver the fresh medium with antimicrobial agent to the biofilm. Perfusing biofilms with media via the dilution vessel into which drugs were added allows biofilm cells to be exposed to exponentially decreasing concentrations of antifungal agent. Once the biofilms reached a steady state, the drugs were added directly to the dilution vessel. The dilution rate of the drug was similar to their *in vivo* half-lives and the volume of the medium in the dilution vessel was calculated from the equation t_1/2_ = 0.6931 V/r, where r is the rate of flow of medium and V is the volume of medium, as the reaction follows first-order decay kinetics [16]. The half-lives of the antifungal agents used in these experiments are 24 and 6.5 hours for Amp-B and VCZ respectively [19,20]. The flow rate was kept at 10 ml/h throughout all experiments.

### Evaluation of the antifungal activity of Amp-B and VCZ on mature biofilms and biofilm-dispersed cells of *C. albicans*

The biofilm device was used to study the effects of Amp-B and VCZ on mature biofilms and the biofilm dispersed cells of *C. albicans* on vascular catheter (Peripheral venous catheter BD Angiocath, reference number 382259, Becton, Dickinson and Company, USA). Briefly, 24 hours old culture of *C. albicans* on Sabouraud Dextrose Agar was used to inoculate RPMI medium adjusted to pH 7 by using MOPS (3-(N-morpholino) propansulfonic acid). The initial inoculum size was standardized in the medium to give 1–5 X 10^6^ CFU/ml. The suspension was then continuously delivered to the device at 30 ml/h for 2 hours. The planktonic (free) cells were removed by delivering sterile RPMI at the same rate for another 2 hours. The biofilms on the catheter were kept under continuous flow of fresh RPMI medium at 10 ml/h. Amp-B and VCZ were tested against 2, 5 and 10 day-old biofilms at initial concentrations equal to their C_max_, 2 and 3 μg/ml respectively [18,19]. The rate of delivery of the drugs and the plain medium was kept at 10 ml/h and by this way the drugs delivered to the biofilms were exponentially diluted at a rate that follows first order kinetics. Four other doses of Amp-B and VCZ were added to the dilution vessel every 24 and 12 hours respectively. One milliliter portions of the effluent samples from the device were taken for cell count (dispersed cells) 24 and 12 hours after addition of each dose of the antifungal agents respectively. After 48 h of the last dose, the catheters were aseptically removed from the device and cut to 1 cm segments. The part of the catheter which is proximal to the medium entry, about 1 cm length between Port 1 and 2, was excluded in further assaying of the biofilm because the medium is allowed to flow out the device from port#2 before reaching this part. All other segments were transferred to test tubes containing 1 ml saline and the adherent cells were dislodged by sonication in aquasonic device, vortexed for 1 minute and counted by trypan blue exclusion and viable count on Sabouraud Dextrose Agar. Drug-free experiments were used as controls where the effect of the antifungal agents on the dispersed cells from and the viable cells within the biofilms were calculated compared to the positive controls.

### Scanning electron microscopy

The Scanning Electron Microscope was used to visualize the biofilm of *C. albicans* on the catheters after 24 hours perfusion with the medium. Catheters, on which the biofilm was formed as described above, were aseptically cut to 1 cm segments and prepared for Scanning Electron Microscope examination as previously described [21]. Briefly, they were fixed in glutaraldehyde in 0.1 M cacodylate buffer containing 0.15 ruthenium red for 3 h at 4°C. The segments were then rinsed in fresh 0.1 M cacodylate buffer for 10 min (repeated three times) and post-fixed in 1.5% osmium tetroxide for 1 hour. They were dehydrated in a series of aqueous ethanol solutions (30–100%) and dried by a critical point dryer (Autosamdri) with CO_2_. The specimens were mounted on aluminium stubs with silver paste, allowed to dry for 3 hours and then coated with gold/palladium using a cool-sputter coater E5100 II (Polaron Instruments). The segments were then examined in the electron microscope (S-500; Hitachi) at 20 kV.

One milliliter portions containing dispersed cells were collected from the 2-day old biofilms before adding the antifungal agents (control), and before adding the third dose of Amp-B or VCZ. The samples were centrifuged at 10,000 rpm for 30 minutes and the sediments were collected, washed with normal saline, fixed in glutaraldehyde in 0.1 M cacodylate buffer containing 0.15 ruthenium red for 3 hours at 4°C and examined as previously described.

### Statistical analysis

Each experiment was performed in quadruplicate and the mean and the Standard Deviation (S.D.) were calculated. One-way analysis of variance (ANOVA) was used to determine the differences between various treatments. Tukey’s pair comparison test was used at the chosen level of probability (*P* < 0.05) to determine significance difference between means.

## Results

### *In vitro* susceptibility of *C. albicans* to Amp-B and VCZ

The MIC of VCZ (0.0325 μg/ml) was eight fold less than that of Amp-B (0.25 μg/ml). The MFC of Amp-B was 0.50 μg/ml while VCZ (a fungistatic agent) was not able to kill the fungus at the maximum tested concentration (64 μg/ml).

### Evaluation of the antifungal activity of Amp-B and VCZ on biofilm-dispersed cells of *C. albicans*

**Figure 2:**
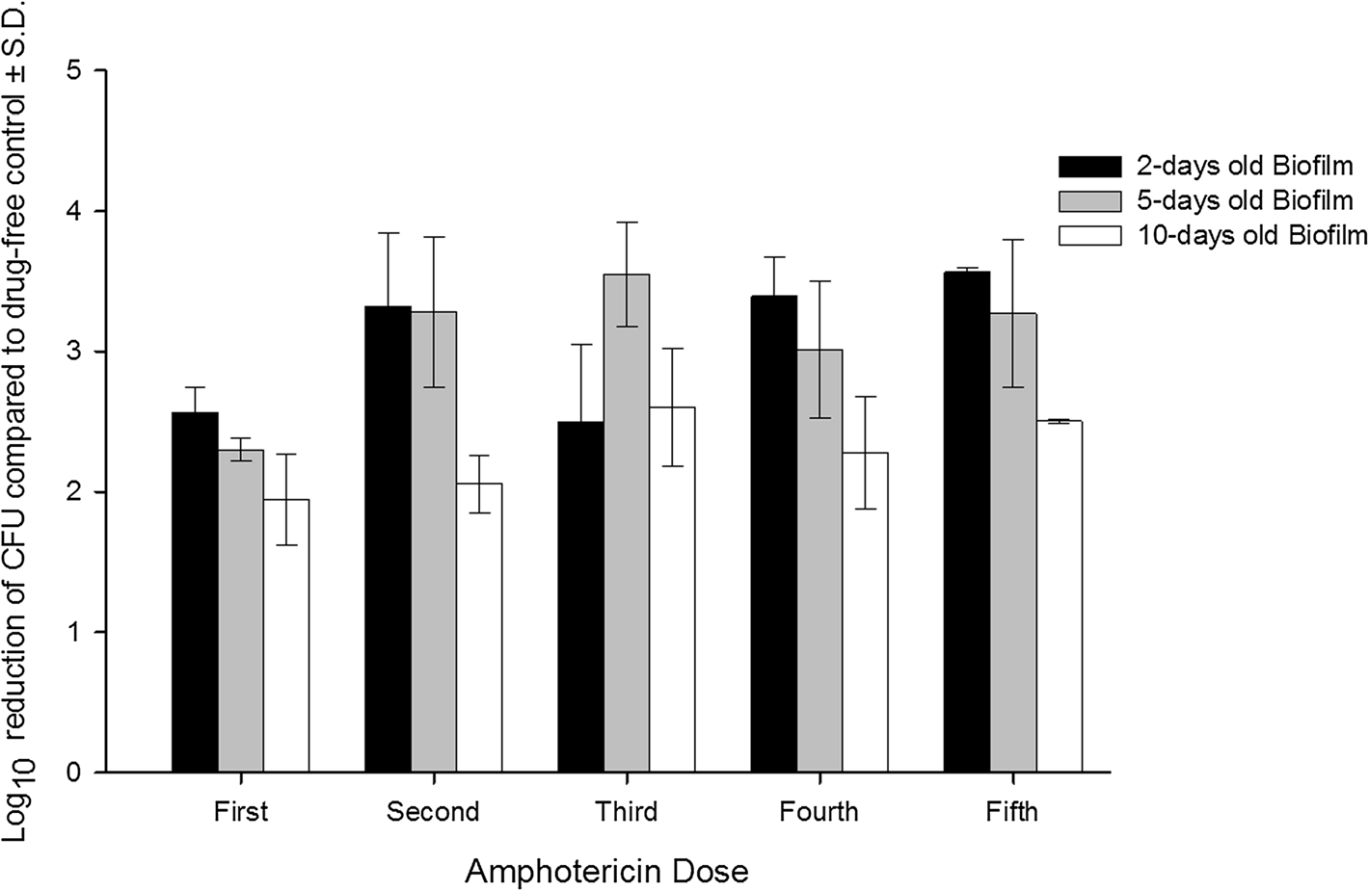
**Activity of amphotericin B on the dispersion of cells from mature biofilms of** ***C. ablicans*** **following addition of 5 doses of the antifungal agent.** The doses were added at 24 hours intervals and the effluent samples, 1 milliliter portions, were collected after 24 hours of addition of each dose.

**Figure 3:**
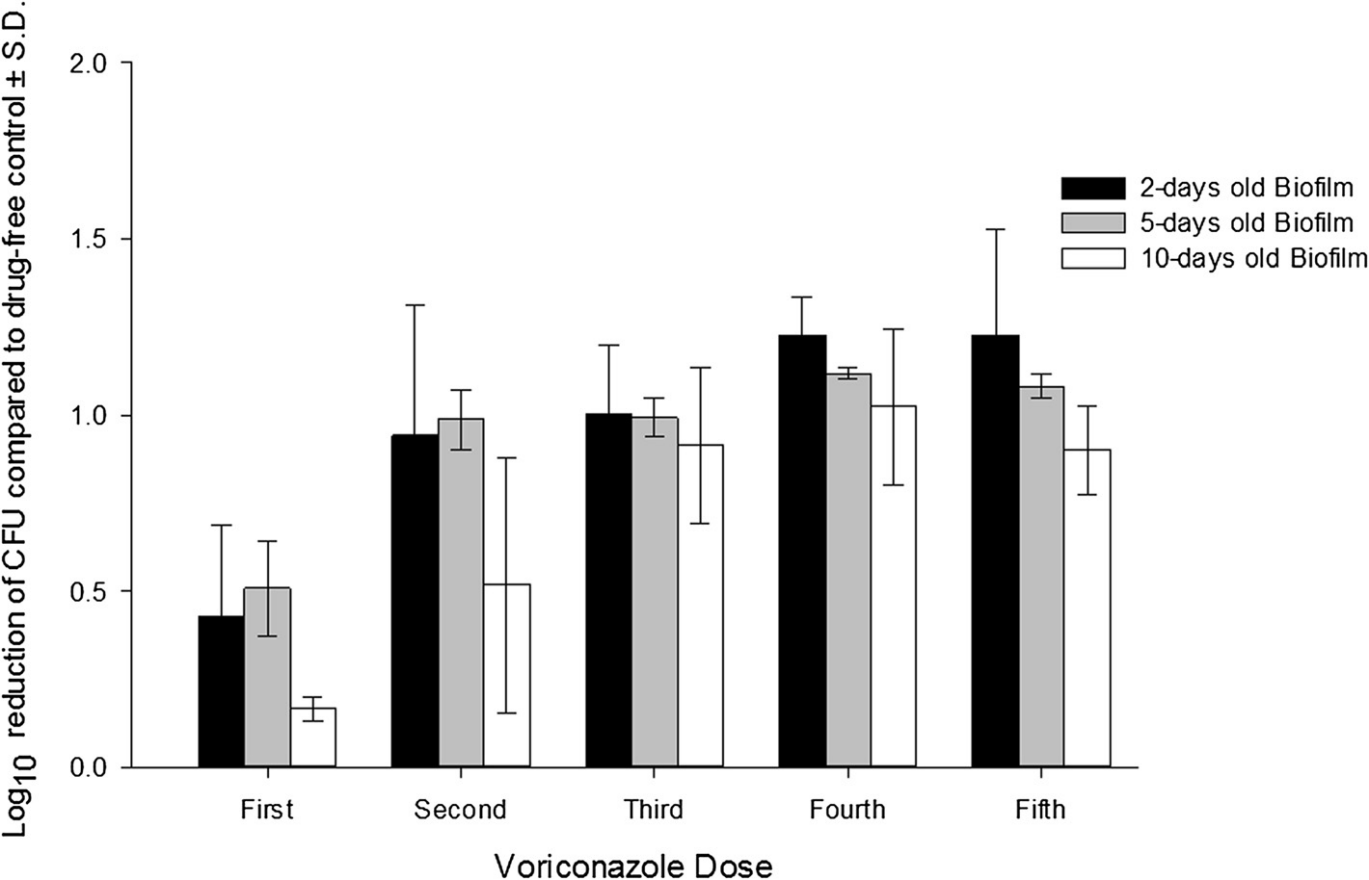
**Activity of voriconazole on the dispersion of cells from mature biofilms of** ***C. ablicans*** **following addition of 5 doses of the antifungal agent.** The doses were added at 12 hours intervals. The effluent samples, 1 milliliter portions, were collected after 12 hours of addition of each dose.

The dispersed cells from the biofilm were quantified before adding each dose of the antifungal agents. Amp-B significantly (p < 0.05) reduced the number of dispersed cells of *C. albicans* from the biofilm (Figures 2 and 3). It was noted that with exponentially decreasing exposure of the biofilms to Amp-B or VCZ, the older the biofilm, the less was the reduction in the number of dispersed cells from it. The maximum log_10_ reduction in the number of eluted cells from the biofilms occurred after the second dose of the antifungal agents, and the reduction values not significantly changed after exposure to the remaining three doses (p > 0.05). When 5 doses of Amp-B were added to 2-day old biofilm, the dispersed cells were reduced by 2.54-3.54 logs. The log_10_ reduction was 2.30-3.55, and 1.94-2.50 following addition of Amp-B to 5- and 10-day old biofilms. On the other hand, VCZ did not show such activity against the dispersed cells. The log_10_ reduction was 0.43-1.22, 0.50-1.11 and 0.16-1.02 when 5 doses of VCZ were added to 2-, 5- and 10-day old biofilms respectively.

### Evaluation of the antifungal activity of Amp-B and VCZ on the mature biofilms of *C. albicans*

**Figure 4:**
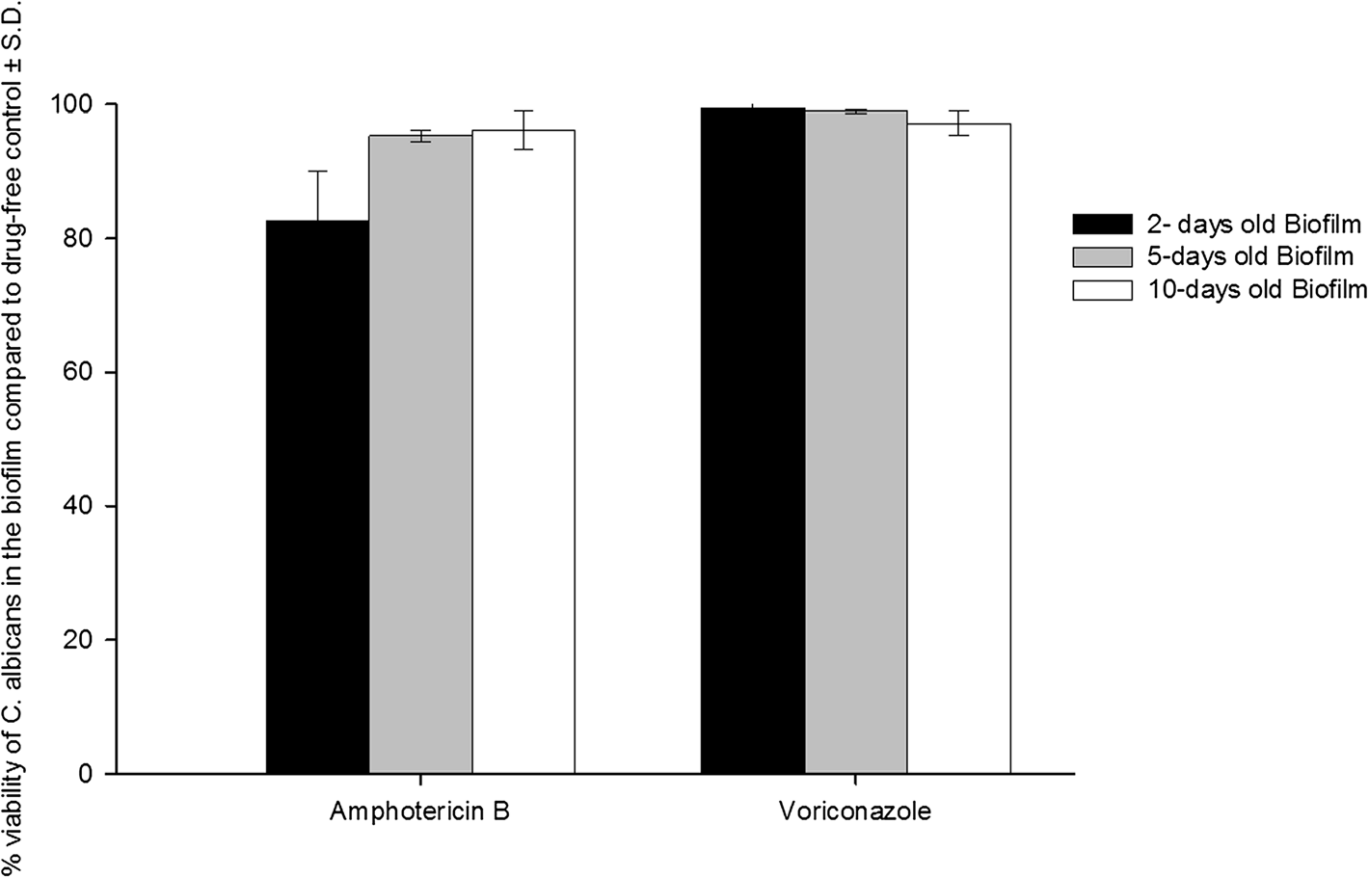
**The antifungal activity of amphotericin B and voriconazole against mature biofilms of** ***C. albians*** **on IV vascular catheter following addition of 5 doses of the antifungal agents.** The viable cells within the biofilm were determined after 48 hours of the last dose and calculated as colony forming unit (CFU) per 1 cm of the catheter segment.

The antifungal agents were tested against 2-, 5-, and 10- day old biofilms of *C. albicans* in the pharmacokinetic biofilm model (Figure 4). Adherent cells were evaluated by viable count of the released cells following sonication of the biofilms. The biofilms of *C. albicans* were resistant to the antifungal drugs. Amp-B was more active against the 2-day old biofilms compared to the effect on 5- and 10-day old biofilms. Following addition of 5 doses to 2-day old biofilms, Amp-B reduced the number of the viable cells within the biofilm by 18% (±7.63) compared to untreated biofilms (drug-free controls), while the reduction was 5 and 4% following addition of the antifungal agent to 5- and 10-day old biofilms. VCZ, on the other hand, showed no significant effect on the viability of *C. albicans* within the tested biofilms after addition of 5 doses of the drug compared to drug-free controls.

### Visualization of the architecture of mature biofilms of *C. albicans* and the effect of Amp-B and VCZ on biofilm dispersion using Scanning Electron Microscopy

**Figure 5:**
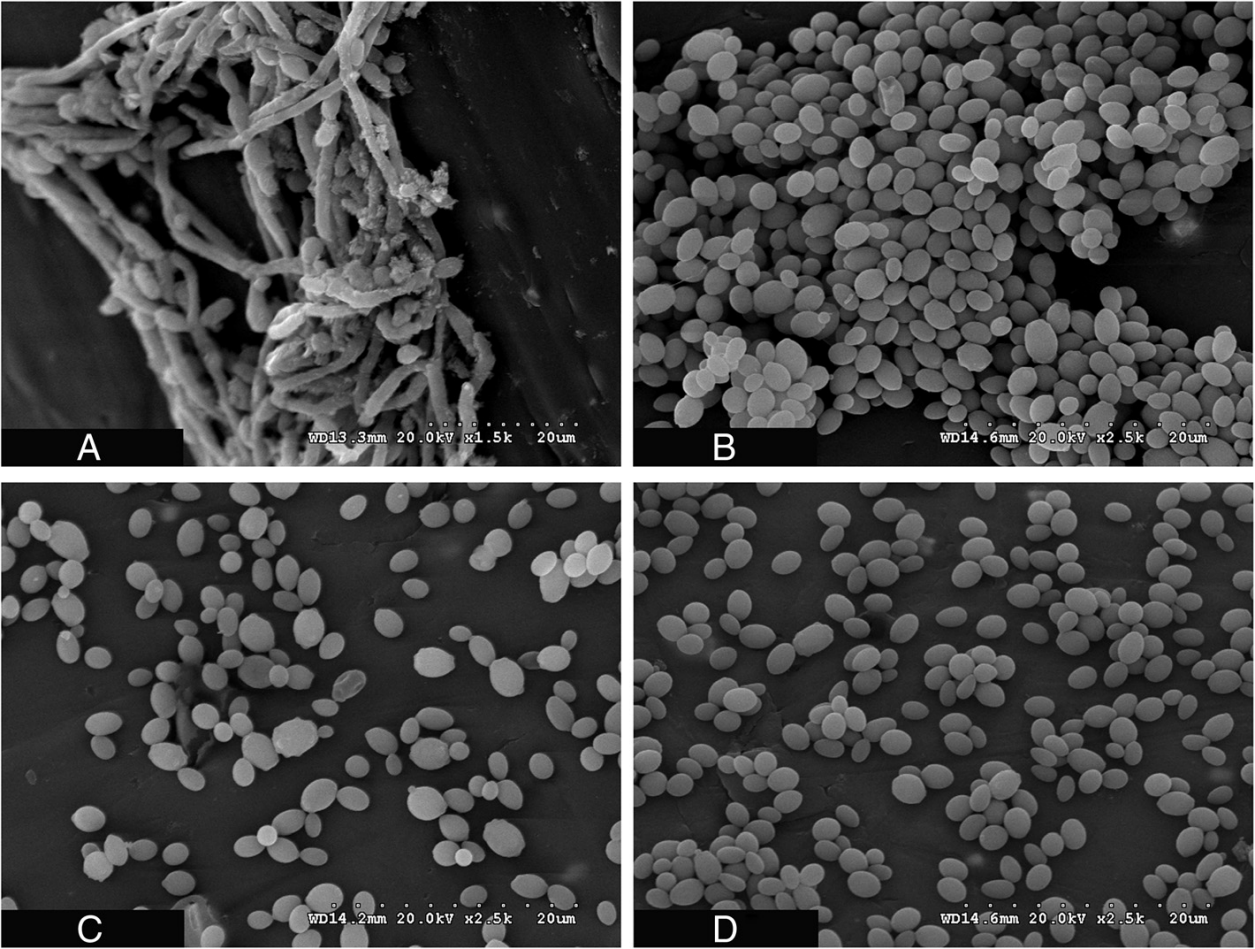
**Scanning Electron Micrograph (SEM) shows. (A)** the architecture of 24 hours old biofilm of *C. albicans* on IV vascular catheter following continuous perfusion of the biofilm with fresh medium. **(B)** The dispersed cells collected from 1 milliliter effluent sample from the biofilm of *C. albicans* on vascular catheter following 2 day of continuous perfusion by fresh medium. **(C)** The dispersed cells collected from 1 milliliter effluent sample from 2-day old biofilm of *C. albicans* on vascular catheter following addition of 2 doses of amphotericin B at 24 hours intervals. **(D)** The dispersed cells collected from 1 milliliter effluent sample from 2-day old biofilm of *C. albicans* on vascular catheter following addition of 2 doses of voriconazole at 12 hours intervals. Note that in the biofilm, the cells exist in the yeast and the long tubular hyphal forms while the dispersed cells exist as yeast cell form only.

The scanning electron micrograph (SEM) of 24 hour old biofilm shows the typical pattern of *C. albicans* cells in the biofilm which consists of yeast form cells (blastospores) and long tubular hyphal cells (Figure 5A). On the other hand, the SEM shows that the dispersed *C. albicans* cells from the biofilms were in yeast form only (Figure 5B, C and D). The SEM was used, semi quantitatively, to compare the number of dispersed cells of *C. albicans* following treatment of the biofilms with 2 doses of Amp-B or VCZ compared to drug-free sample. The reduction in the number of dispersed cells was obvious after treating the biofilms of *C. albicans* with 2 doses of Amp-B compared to VCZ.

## Discussion

Candida infection associated with biofilm formation is a serious nosocomial problem. The resistance of C. *albicans* biofilms enables the organism to escape the immune response as well as the antifungal drug activity [22].

Biofilm-associated microbial infection in humans is widespread and increasing, and occurs largely as a consequence of the increase in the use of indwelling medical devices, such as intravenous and urinary catheters, pacemakers and prosthetic joints [23]. Recent evidence suggests that the majority of disease produced by *C. albicans* is associated with biofilm formation [10].

In this study, a newly developed *in vitro* pharmacokinetic biofilm model (that simulates the *in vivo* conditions) in which microorganisms are exposed to supra and sub MIC of the drugs was used. The pharmacokinetic model simulates the serum antibiotic concentration-time curve obtained during intermittent administration of the antifungal agents in clinical practice. These experimental conditions can be achieved by a dilution method in which the reactions follow first order kinetics [16,17,24]. The novel biofilm device was constructed to fit the conditions of the experiment. Its design makes it easy to contain a full length catheter in the main chamber instead of using segments or piece of plastics as in other available devices. The device can also be adjusted to different configurations to fit the experimental conditions, static or dynamic system, required to simulate and study interaction between microbial biofilms and antimicrobial agents.

Intravenous catheter was selected as a model of the indwelling device to study antifungal activity of the two drugs on the biofilms of *C. albicans*. Catheter-related infections are the major cause of morbidity and mortality among hospitalized patients, and the biofilms on catheters are associated with 90% of these infections [11]. Catheter-associated Candida biofilms can lead to candidemia with an approximate incidence of one case per 100 hospital admissions [25]. Biofilm-associated Candida infections cause mortality rates as high as 30%, with annual cost of antifungal therapy estimated at US$2.6 billion in the United Sates [26,27].

For this study, we chose to compare Amp-B, a conventional broad spectrum antifungal agent, with VCZ, a representative of the triazole class, with the aim of evaluating our *in vitro* model. Both drugs are not first line therapy in invasive Candida infection. Treatment of candidemia with fluconazole, first line therapy, and Amp-B have demonstrated comparable results [28,29]. VCZ, on the other hand, has shown equivalent or even better activity against Candida species compared to fluconazole [30-32]. The antifungal activity of Amp-B and VCZ was evaluated by measuring their effect on the dispersed cells from the biofilm and the viability of the cells within the biofilm following the exposure to 5 doses of the antifungal agents. It was noted that with incrementally decreasing exposure of the biofilms to the drugs, there was declining reduction in the number of dispersed cells from older biofilms (Figures 2 and 3). The maximum log_10_ reduction in the number of eluted cells from the biofilms occurred after the second dose of the antifungal agents, and changed insignificantly upon exposure to the remaining three doses (p > 0.05). In a pharmacokinetic study conducted by Gander et al. [17], linezolid at exponentially decreasing concentrations was tested against the dispersed cells from staphylococcal biofilms. They found that the maximum log_10_ reduction in the dispersed cells was observed after the first dose. When a biofilm is at steady state, cells are shed from it at a constant rate, which could be considered as the normal rate. The biofilms in this model were exposed first to concentrations above the MICs and then to subinhibitory concentrations of the drugs. Subinhibitory concentrations of antimicrobial agents often influence the growth rate of the microorganisms [33].

The number of cells dispersed from a biofilm decreases when the biofilm is exposed to an inhibitory concentration of a drug, and increases on removal of the drug, eventually returning to the original steady state [16]. Dispersed cells of *C. albicans* from biofilm to circulation may cause biofilm-associated candidemia. These dispersed cells have been found to have a different pattern of antimicrobial susceptibility from the same cells that exist in the biofilm or planktonic phase of growth [34]. In our model, Amp-B significantly (p < 0.5) reduced the number of dispersed cells from the biofilm compared to drug-free control but could not completely stop the dispersion process. VCZ, on the other hand, was less active in reducing dispersed cells.

Amp-B significantly (p < 0.05) reduced the number of the viable cells within the 2-day old biofilms compared to control samples (Figure 4), This effect was not observed with the 5- and 10-day old biofilms. VCZ showed no effect on the viability of C*. albicans* within all tested biofilms. It has been reported that the progression of drug resistance within Candida biofilms is associated with a parallel increase in the maturation process [35]. Biofilm cells typically have very slow growth rates and are surviving under nutrient limitation compared to planktonic cells grown in batch cultures *in vitro* [16]. Other mechanisms have been postulated to explain recalcitrance of the biofilm to antimicrobial agents including contact-dependent gene expression, interaction with the extracellular polymeric matrix, and poor penetration through the biofilm mass [14,21].

The scanning electron micrograph (Figure 5A) shows that the biofilm of *C. albicans* consists of blastospores (yeast form) and long tubular hyphal cells. *C. albicans* biofilms grown *in vitro* often have a foundation of yeast cells from which a hyphal layer originates [1]. In general, the Candidal biofilm consists of a dense network of yeast cells, pseudohyphae, and hyphae, which are very difficult to eradicate by antifungal agents [21].

It would be expected that the older the biofilm, the more complex and dense the network of the yeast cells, which in turn would make the biofilm more recalcitrant by acting as a barrier for the antifungal agents. Unlike the cells in the biofilm, dispersed cells of *C. albicans* appeared in yeast form (Figure 5B, C and D). It has been reported that *C. albicans* biofilm dispersion is dependent on growing conditions, and that the dispersed cells are mostly in the yeast form [36]. The SEM supports our finding that Amp-B was capable of reducing the dispersion of the *C. albicans* from the biofilms but not to the extent of stopping it.

## Conclusion

One clinical isolate of *C. albicans* was used to validate the biofilm device and to study the antimicrobial activity of the selected antifungal agents against the biofilm and the cells dispersed from it. The antifungal activity of both antifungal agents was inefficient against *C. albicans* cells that grew in or dispersed from the biofilms in the constructed model. The model mimics the conditions *in vivo* when the biofilm is formed, dispersed and treated following exposure to the antifungal agents at concentrations that decrease exponentially similar to the intermittent administration in clinical practice. Amp-B, a conventional broad spectrum antifungal agent, and VCZ, a model of triazole antifungals, failed to stop the dispersion of *C. ablicans* cells or to eradicate the biofilm of the yeast cells. Our results support the clinical finding that the formation of biofilms inside an implant device leads to failure of the device and acts as a source of refractory infections. It is difficult to treat the implant-associated infection not only due to failure of the antimicrobial agents to kill the cells within the biofilm but also to stop dispersion of cells that leads to dissemination which necessitates the removal of the device.

This is the first study which investigates the effects of antifungal agents on the biofilm and biofilm-dispersion of *C. albicans* in an *in vitro* pharmacokinetic biofilm model.
